# Exploring phytochemistry, antioxidant potential, essential oil profiling and bioactive profiling of *Pogostemon mollis* Benth. through GC–MS and UPLC-QTOF-MS/MS

**DOI:** 10.1038/s41598-026-35989-2

**Published:** 2026-01-27

**Authors:** Sobiyanaz Momin, Mayur Jadhav, Rajaram Gurav

**Affiliations:** Department of Botany, Shivaji University, Vidyanagar, Kolhapur, 416004 India

**Keywords:** Antioxidant, Essential oil, GC–MS, Lamiaceae, *Pogostemon mollis*, UPLC-QTOF-MS/MS, Biochemistry, Biotechnology, Chemical biology, Chemistry, Drug discovery, Plant sciences

## Abstract

The genus *Pogostemon* (Lamiaceae) is known for its diverse pharmacological and ethnomedicinal properties, which may be due to the presence of various bioactive constituents. This study investigates antioxidant potential, profiling of phytochemicals, essential oil and their derivatives from *Pogostemon mollis* Benth., and correlation among them. Plant parts were taken in fresh and dried forms, were extracted using methanol, acetone and distilled water through ultrasonication extraction method. Essential oil was obtained from the aerial parts through hydro-distillation with a Clevenger apparatus. Antioxidant activity by DPPH, FRAP, and ABTS, demonstrating significant radical scavenging and reducing power; dry aqueous leaf extract showing the highest inhibition (65.1 ± 0.11%) and the lowest in fresh aqueous stem extract (9.80 ± 0.24%). Total phenolic and flavonoid contents were checked for extracts, revealing maximum phenolic content in dry methanolic leaf extract (249.9 ± 0.47 mg GAE/g) and minimum in fresh acetonic root extract (64.12 ± 0.31 mg GAE/g). The highest flavonoid content was found in dry acetonic leaf extract (50.5 ± 0.00 mg RE/g), while the lowest was in fresh aqueous root extract (16.04 ± 0.49 mg RE/g). UPLC-QTOF-MS/MS revealed 99 bioactive compounds, such as anticancer (camptothecin), antiviral (zidovudine), flavonoid (luteolin), and terpenoid (nerolidol) agents. GC–MS identified 68 compounds in the essential oil, with major constituents including lupeol (6.33%), alpha-cyperone (7.15%), and caryophyllene oxide (7.23%). Significant correlations were found between total phenolic content, total flavonoid content, and antioxidant activities, highlighting the therapeutic potential of *P. mollis* for developing natural antioxidants and bioactive formulations.

## Introduction

The Lamiaceae family includes numerous aromatic species widely utilised in traditional medicine, as well as in pharma and food sectors, due to diverse biological activities^1^. A total of 272 genera of flowering plants were identified as having medicinal uses, among which approximately 4% of studied species belonged to the Lamiaceae family^2^. *Pogostemon mollis* is an undershrub that grows to a height of 30–60 cm that growing on hills above 1200 m on exposed rocks and bare slopes. It has been found predominantly in various parts of southwestern India, as well as in the Western and Eastern Ghats. It contains bioactive constituents that have been used since ancient times and are associated with various therapeutic effects, such as pain relief, asthma management, anticancer activity, inflammation reduction, and antimicrobial action^3^. The therapeutic potential of these plant species is attributed to their diverse phytochemical profile, including several secondary metabolites such as phenolics, flavonoids, alkaloids, and other groups, which exhibit notable bioactivity as antioxidants, antimicrobial agents, and anti-pathogenicity,thereby supporting their traditional use in disease management^3–5^. George et al.^6^ studied ethyl acetate extract of *P. mollis,* having higher phenolic concentration of 474.8 mg GAE/g, followed by methanol 311.1 mg GAE/g and acetone extracts 309.8 mg GAE/g. DPPH radical scavenging reveal that the ethyl acetate extract had 3.1 µg/mL, while the acetone extract had 3.8 µg/mL. It exhibited maximum reduction in nitric oxide levels at 11.7%, showcasing its potential in scavenging reactive nitrogen species. Flavonoids possess potent antioxidant, anti-inflammatory, anticancer and antimicrobial activities^7^. Saranya et al.^8^ studied qualitative screening of *P. mollis* with solvents (petroleum ether, ethyl acetate, and ethanol) for the different parts, as leaf, stem, and root. Results showed a significant presence of secondary metabolites, indicating the plant’s potential for therapeutic applications. Analysis of ash values indicated a high purity level in plant extracts. Total ash, along with its water-soluble and acid-insoluble fractions, was measured to assess inorganic content, which is essential for assessing the quality and authenticity of herbal medicines^9^. Earlier, Li^10^ and Chakrapani et al.^11^ have reported many active compounds in *P. mollis*, through HPLC; George et al.^6^ found several compounds in ethyl acetate, methanol and acetone extract. Muthuraj et al.^9^ detected 47 bioactive compounds from the methanolic extract by GC–MS. The essential oil of the *Opopanax* genus exhibits low yield but a chemically diverse profile dominated by monoterpenes and sesquiterpenes, as reported in Turkish populations. Babacan et al.^12^ identified major constituents such as trans-β-ocimene, myrcene, α-pinene, and germacrene D, highlighting the genus’s phytochemical and pharmacological relevance.

Kurt-Celep et al.^13^ reported that extracts of *Astragalus caraganae* possess a rich profile of phenolic and flavonoid constituents, with compounds such as rutin, p-coumaric acid, chlorogenic acid, isoquercitrin, and delphinidin-3,5-diglucoside predominating. Their study demonstrated notable antioxidant and enzyme-inhibitory activities, along with non-cytotoxic yet concentration-dependent cytostatic effects on HDF cells, underscoring the plant’s pharmacological relevance. Yagi et al.^14^ showed that *Phlomis fruticosa*, *P. herba-venti* and *P. kurdica* possess diverse bioactive metabolites, with methanol extracts providing the richest chemical profiles. Their study demonstrated notable antioxidant, metal-chelating and cholinesterase-inhibitory activities across the species.

Essential oils are a class of volatile, aromatic compounds having broad applications in both culinary and perfumery sectors. These oils typically consist of intricate blends of terpenes—mainly monoterpenes and sesquiterpenes—and their corresponding oxygenated compounds^15^. Evaluation of antimicrobial properties of essential oil from *P. mollis* yielded significant findings regarding its effectiveness against various bacterial and fungal strains. It has antifungal activity against *Staphylococcus aureus, Streptococcus pyogenes* and *Escherichia coli*, and antimicrobial action against *Candida tropicalis*, *Candida albicans Proteus mirabilis*^9,16^)*.* The SARS-CoV-2 viral infection, which emerged in late 2019, rapidly spread worldwide and was declared a global pandemic, with ongoing impact to the present day. Secondary metabolites have been explored for their interactions with various target proteins of SARS-CoV-2 in search of potential lead compounds against COVID-19. Use of in silico tools has allowed researchers to explore a vast range of medicinal plant compounds, effectively narrowing down bioactive leads and reducing the reliance on traditional experimental methods^17^. *Pogostemon cablin* has been explored for its potential antiviral properties, including its effectiveness against the COVID-19 virus^18^. MTT assay revealed that extracts exhibited cytotoxic activity against RAW 264.7, MCF-7, and Caco-2 cell lines. Extracts exhibited a dose-dependent reduction in cell viability^6^.

**Fig. 1:**
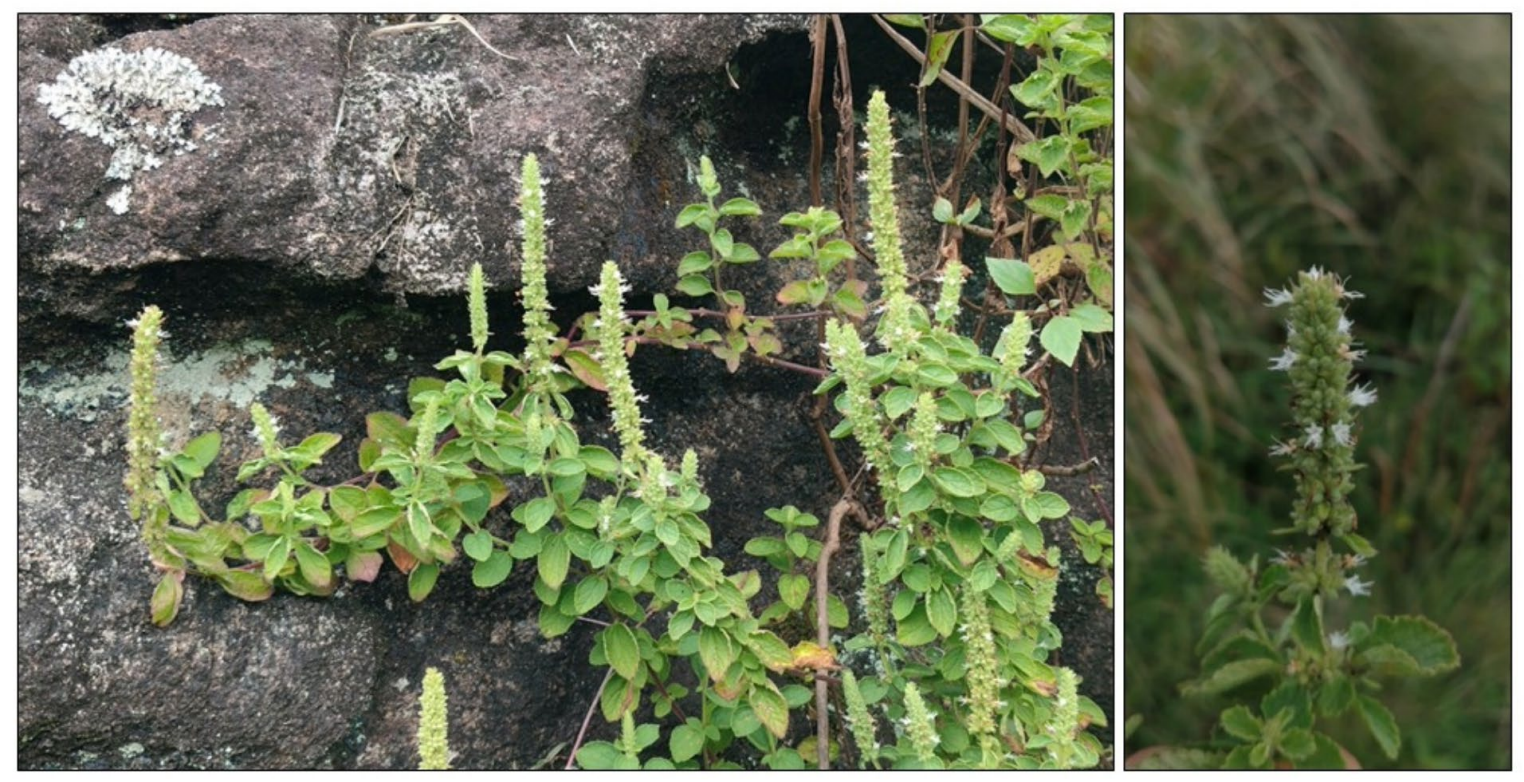
*Pogostemon mollis* Benth. (**A**) Habitat, (**B**) Inflorescence.

Earlier studies on *Pogostemon mollis* have mainly focused on either single solvent extracts or limited plant parts. Previous investigations did not provide a comprehensive comparison of leaf, stem, and root extracts nor evaluate how different solvents (water, methanol, acetone) influence the phytochemical profile, essential oil composition, and antioxidant activity. The present study therefore offers the first detailed, organ-wise and solvent-wise evaluation of *P. mollis*, providing a more complete understanding of its chemical diversity and supporting its traditional medicinal applications (Fig. 1).

## Materials and methods

### Collection and authentication

*Pogostemon mollis* Benth. collected from Trivandrum district, 8°45′36.39″N, 77° 6′ 39.52″E, Ponmudi Hills, Kerala. Plant specimens were taxonomically identified and authenticated by Prof. Rajaram Gurav. Specimens from the same population were deposited as voucher specimens (SAM-006, SAM-007) in the Herbarium of Shivaji University (SUK). All necessary permits for the collection and use of plant materials were obtained from the Kerala Forest Department (No. KFDHQ/4512/2024-CWW/WL10).

### Preparation of plant extracts and essential oil

The biochemical, antioxidant activities of fresh and dried leaf, stems, and roots of *P. mollis* were studied. Extract prepared by the Ultrasonication method using methanol, acetone, and distilled water as solvents^19^. Specimens were processed as both fresh and oven-dried,the drying occurred at 60 °C, and fresh samples had been frozen at − 80 °C for an ongoing study. Essential oil isolated from aerial parts (leaf, inflorescence with floral buds) through hydro distillation was performed using a Clevenger-type apparatus^20^. Subsequently, oil was stored at 4 °C until it was required for continued use.

### Antioxidant potential 

#### DPPH radical scavenging activity

Radical scavenging potential was evaluated using the protocol of Aquino et al.^21^, with minor modifications. In brief, 10 μl of extract was taken and mixed with 290 μl freshly prepared DPPH solution. The components were mixed uniformly and kept at room temperature for incubation for over 30 min. After decolourization, the reaction mixture was measured at 517 nm. A standard curve was constructed using ascorbic acid (mg/ml). Results were represented as a percentage of inhibition.

$${\text{Percentage of Inhibition}} = \left[ {\left( {{\mathrm{Abs}}.{\text{ Control}} - {\mathrm{Abs}}.{\text{ Sample}}} \right)/{\mathrm{Abs}}.{\text{ Control}}} \right] \times {1}00$$

#### FRAP (ferric reducing antioxidant power) assay

Reducing power assay was determined by following the Benzie and Strain et al.^22^ method with a few changes. To prepare the FRAP reagent solution, 0.3 M acetate buffer (pH 3.6), 10 mM TPTZ in 40 mM HCl, and 20 mM FeCl₃·6H₂O were mixed in a 10:1:1 volume ratio. The resulting solution was then incubated in a water bath at 37 °C for 10 min before use. In this experiment, a 10 μl volume of plant extract was mixed with 290 µl freshly prepared FRAP solution, and following a 30-min incubation in the dark, absorbance was recorded at 595 nm. Values were calculated and expressed as milligrams of ascorbic acid equivalent per gram (mg AAE/g) of sample weight.

#### ABTS (2, 2-azino-bis-3-ethylbenzothiazoline-6-sulphonic acid) assay

ABTS is extensively applied in antioxidant activity assessment or free radical scavenging capacity of plant extracts. The assay was performed following the method described by Re et al.^23^. For the assay, 10 μl of the plant extract was mixed with 190 μl of ABTS working solution. The ABTS reagent was prepared by mixing equal volumes of a 7 mM aqueous solution of ABTS and a 2.45 mM aqueous solution of potassium persulfate, and incubating this mixture for 12–16 h in the dark. The resulting solution was then diluted to achieve an absorbance of 0.70 ± 0.02 at 734 nm. After mixing with the plant extract, the mixture was kept at room temperature for 10 min. Antioxidant activity was assessed by measuring the reduction in absorbance at 734 nm. Ascorbic acid served as a reference standard. The percentage of free radical scavenging or inhibition was calculated accordingly.

$${\text{ABTS scavenging activity }}\left( \% \right) = \left[ {\left( {{\mathrm{Abs}}.{\text{ Control}} - {\mathrm{Abs}}.{\text{ Sample}}} \right)/{\mathrm{Abs}}.{\text{ Control}}} \right] \times {1}00$$

### Quantitative phytochemistry 

#### Determination of total phenolic content

TPC was estimated using the Folin-Ciocalteu (FC) reagent following the modified protocol of Singleton and Rossi^24^. In brief, 125 µl of extract was combined with 1.8 ml of FC reagent and incubated at 25 °C for 5 min. Subsequently, 1.2 ml of 15% Na_2_CO_3_ was added, mixture was allowed to react for 90 min room temperature. Absorbance was measured at 765 nm, and total phenolic content (TPC) was calculated in milligrams of gallic acid equivalent per gram of sample (mg GAE/g) utilizing a standard curve for calibration.

#### Determination of total flavonoid content

Flavonoid quantification was carried out following the colorimetric procedure of Luximon-Ramma et al.^25^. To quantify total flavonoid content (TFC), 150 μl of plant extract was combined with 150 μl of a 2% aluminium chloride solution and incubated at room temperature for 10 min. Absorbance was recorded at 367 nm, and TFC was expressed as milligrams of rutin equivalents per gram of sample (mg RE/g).

### Bioactive Compound Profiling (UPLC Q-TOF MSMS)

Analysis was performed using ultra-performance liquid chromatography coupled with quadrupole time-of-flight tandem mass spectrometry (UPLC-QToF-MS/MS) was conducted through an Agilent Q-ToF G6540B mass spectrometer integrated with an Agilent 1260 Infinity II HPLC system. Chromatographic separation was carried out on an Agilent Eclipse XDB-C18 column (3.0 × 150 mm, 3.5 µm particle size), maintained at a constant temperature of 40 °C. Mobile phase comprised Solvent A (0.1% formic acid in water) and Solvent B (0.1% formic acid in acetonitrile), delivered at a flow rate of 0.3 mL/min. Gradient elution was as follows: A gradient elution program was employed over a total runtime of 30 min. The initial mobile phase composition was 95% solvent A and 5% solvent B, maintained from 0.0 to 2.0 min. The proportion of solvent B was then linearly increased to 95%, reaching this composition at 25.0 min and held constant until 28.0 min. At 28.1 min, the gradient was rapidly returned to the initial conditions (95% A, 5% B), and the system was re-equilibrated under these conditions for 30 min. The mass spectrometer was operated in dual electrospray ionization (Dual AJS ESI) mode with both positive and negative ionization. Data were collected over a mass-to-charge ratio range from 100 to 1700. The analysis was carried out using the following optimised instrumental conditions: the nebuliser gas temperature was maintained at 300 °C, while the sheath gas temperature was set at 350 °C. The drying gas flow rate was 8L/min, and the sheath gas flow rate was maintained at 11L/min. The nebuliser pressure was adjusted to 35psi. A capillary voltage of 3500 V and a nozzle voltage of 1000 V were applied to ensure efficient ionization and transmission of the analyte.

### Essential oil profiling (GC–MS)

The volatile constituents of *P. mollis* oil were analysed by GC–MS employing the TQ 8050 plus instrument (Shimadzu, Japan) equipped with an HS-20 unit. An SH-Rxi-5Sil MS capillary column (30 m length × 0.25 mm internal diameter × 0.25 μm film thickness) was used for the separation. Injection mode was set to split, with a split ratio of 1.0. Inlet pressure conditions were regulated at 75.2 kPa, and the linear velocity of carrier gas was 41.4 cm/sec. A purge flow of 3.0 ml/min was applied. Column oven temperature was programmed from 50 to 260 °C. High-purity helium gas (99.9%) was employed as carrier gas, maintained at a constant flow rate throughout analysis. Compound identification was carried out by interpreting the mass spectra obtained from GC–MS, comparing them against spectral databases of the National Institute of Standards and Technology (NIST) and WILEY-08 libraries.

### Statistical analysis

All experiments were conducted in triplicate, and the resulting average values were considered as individual data points. Results are presented as the mean ± standard error (SE). Experimental data were statistically analyzed using one-way ANOVA, and significant differences between mean values were determined by Duncan’s Multiple Range Test (p ≤ 0.05) using SPSS software (version 16). The correlation coefficient was determined between TPC, TFC, and antioxidant activities using SPSS (version 16.0). PAST software (version 3.01) was used to perform Principal Component Analysis (PCA) and Hierarchical Cluster Analysis (HCA) to analyse data derived from phytochemical profiling and antioxidant ability of various extracts.

## Results and discussion

### Antioxidant potential

#### 2,2-diphenyl-1-picrylhydrazyl assay (DPPH) assay

**Fig. 2:**
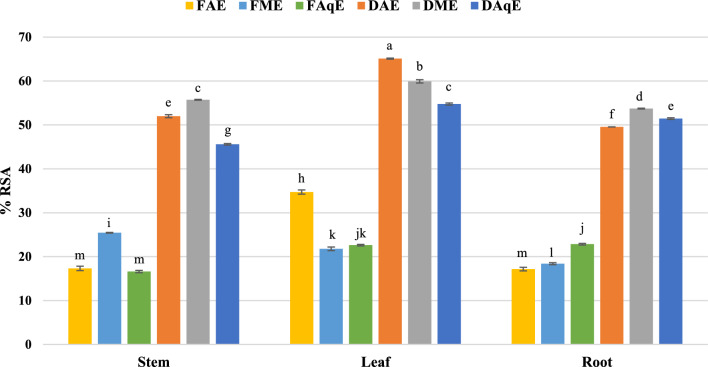
DPPH radical scavenging activity *P. mollis* in different solvents with fresh and dried plant parts (*FAE* fresh acetone extract, *FME* fresh methanol extract, *FAqE* Fresh aqueous extract, *DAE* dry acetone extract, *DME* dry methanol extract, *DAqE* dry aqueous extract).

A colour changes from purple to yellow was observed in the DPPH assay with a decrease in absorbance. Colour change occurs due to donating the hydrogen for scavenging free radicals by antioxidants, which causes a stable form of the DPPH molecule. In the present study, methanol and acetone extracts of both species showed the highest Radical Scavenging Activity (RSA), as presented in (Table 1 and Fig. 2). Dry aqueous leaf extract showed the highest percentage inhibition (65.1 ± 0.11), while the fresh acetonic stem extract % inhibition was minimum (16.62 ± 0.26).

**Table 1:** DPPH, FRAP and ABTS activity of *P. mollis* Benth.

Extraction code	DPPH^α^	FRAP^β^	ABTS^α^
DSAq	52.00 ± 0.33ᵉ	28.01 ± 0.18^ fg^	52.50 ± 0.01^d^
DSM	55.70 ± 0.17ᶜ	31.49 ± 0.13^c^	55.60 ± 0.23^b^
DSA	45.60 ± 0.08ᵍ	26.70 ± 0.06^ g^	51.30 ± 0.10^e^
DLAq	65.10 ± 0.11ᵃ	35.33 ± 0.00^b^	55.70 ± 0.19^b^
DLM	59.90 ± 0.24ᵇ	40.23 ± 0.09^a^	59.40 ± 0.17^a^
DLA	54.80 ± 0.38ᶜ	32.28 ± 0.10^c^	54.50 ± 0.09^c^
DRAq	49.50 ± 0.01ᶠ	29.37 ± 0.02^ef^	48.50 ± 0.01f.
DRM	53.70 ± 0.08ᵈ	28.25 ± 0.01f.	50.20 ± 0.01^e^
DRA	51.40 ± 0.18ᵉ	30.15 ± 0.06^d^	47.40 ± 0.00^ g^
FSAq	17.32 ± 0.50ᵐ	09.80 ± 0.24^ m^	27.30 ± 0.11^ h^
FSM	25.40 ± 0.08ⁱ	17.47 ± 0.27^ h^	22.90 ± 0.27^ l^
FSA	16.62 ± 0.26ᵐ	15.58 ± 0.17^i^	22.40 ± 0.23^ l^
FLAq	34.72 ± 0.47^ h^	13.61 ± 0.18^j^	26.10 ± 0.20^ k^
FLM	21.79 ± 0.42ᵏ	13.27 ± 0.14^jk^	19.70 ± 0.06^ m^
FLA	22.62 ± 0.18ʲᵏ	13.35 ± 0.41^jk^	25.50 ± 0.18^ k^
FRAq	17.16 ± 0.41ᵐ	11.09 ± 0.41^ lm^	26.50 ± 0.22^ij^
FRM	18.39 ± 0.23ˡ	12.61 ± 0.22^jk^	25.70 ± 0.39^ k^
FRA	22.84 ± 0.18ʲ	11.31 ± 0.52^kl^	23.20 ± 0.30^1^

*DSAq* dry stem aqueous extract, *DSM* dry stem methanol extract, *DSA* dry stem acetone extract, *DLAq* dry leaf aqueous extract, *DLM* dry leaf methanol extract, *DLA* dry leaf acetone extract, *DRAq* dry root aqueous extract, *DRM* dry root methanol extract, *DRA* dry root acetone extract, *FSAq* fresh stem aqueous extract, *FSM* fresh stem methanol extract, *FSA* fresh stem acetone extract, *FLAq* fresh leaf aqueous extract, *FLM* fresh leaf methanol extract, *FLA* fresh leaf acetone extract, *FRAq* fresh root aqueous extract, *FRM* fresh root methanol extract, *FRA* fresh root acetone extract.

Values are means of three replicate determinations ± standard error. Mean values in the same column with different alphabets shows statistically significant differences (p ≤ 0.05) according to Duncan’s multiple range test. **α** (% inhibition) and **β** (mg AAE /g extract).

#### Ferric reducing antioxidant power (FRAP) assay

**Fig. 3:**
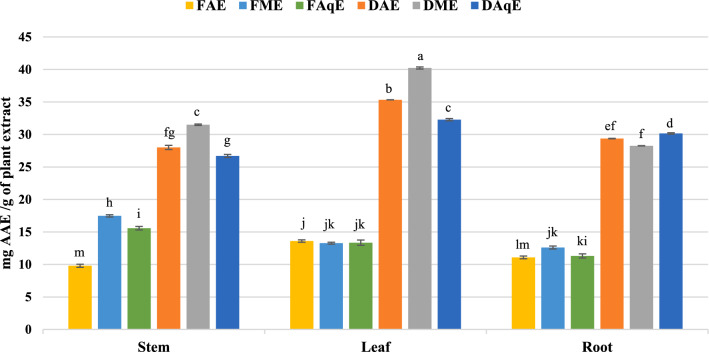
FRAP activity *P. mollis* in different solvents with fresh and dried plant parts. *FAE* fresh acetone extract, *FME* fresh methanol extract, *FAqE* fresh aqueous extract, *DAE* dry acetone extract, *DME* dry methanol extract, *DAqE* dry aqueous extract.

The FRAP assay measures antioxidant activity following the reduction of ferric ions, where the test solution shifts from yellow to green or blue hues, indicating the sample’s reducing power. A compound’s capacity to act as a reducing agent is a key marker of its antioxidant strength. FRAP assay assesses this by monitoring the transformation of ferric (Fe3⁺)-TPTZ complex into its ferrous (Fe2⁺) form, producing a coloured complex indicative of antioxidant activity. Antioxidant capacity is closely associated with the reducing power of bioactive compounds, which reflects their ability to donate electrons. Dry methanolic leaf extract showed the highest FRAP (40.23 ± 0.09) while fresh aqueous stem extract was minimum 9.80 ± 0.24 mg AAE/g (Table 1 & Fig. 3).

#### ABTS assay

**Fig. 4:**
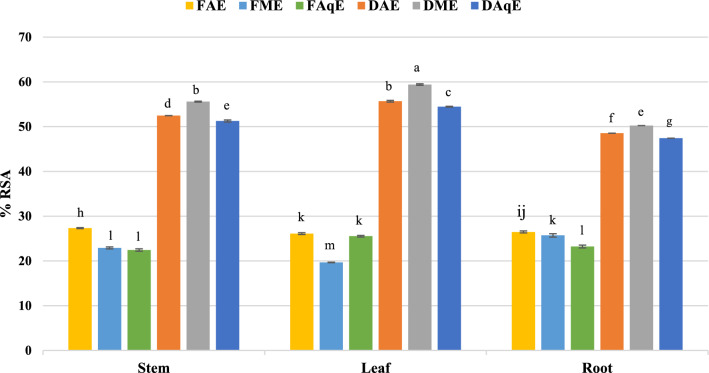
ABTS activity *P. mollis* in different solvents with fresh and dried plant parts. *FAE* fresh acetone extract, *FME* fresh methanol extract, *FAqE* fresh aqueous extract, *DAE* dry acetone extract, *DME* dry methanol extract, *DAqE* dry aqueous extract.

ABTS assay, based on an electron transfer mechanism, was used to assess radical scavenging potential. It involves the reduction of the dark blue ABTS**⁺** cation (2,2′-azino-bis(3-ethylbenzothiazoline-6-sulfonic acid)) by antioxidants into a colourless form. This change can be quantified using a spectrophotometer. Take 10 μl extract, then add 290 μl ABTS, then incubate in the dark for over 30 min at room temperature. Take absorbance at 734 nm. Maximum inhibition was observed in dry leaf extract prepared using methanol (59.4 ± 0.17%), while the fresh methanolic extract of leaf % inhibition was minimum (19.7 ± 0.06) as represented in (Table 1 & Fig. 4).

### Quantitative phytochemistry

#### Determination of total phenolic content

**Fig. 5:**
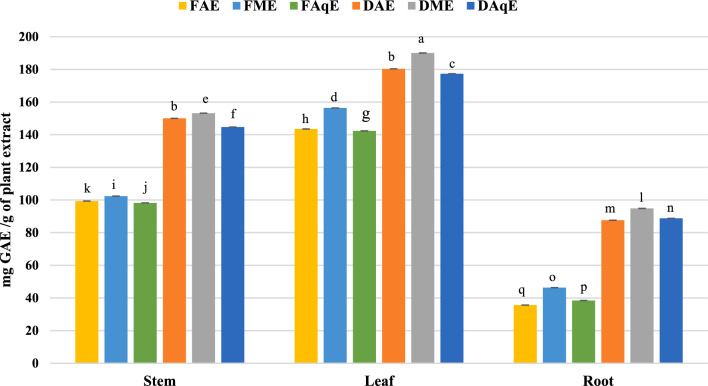
Total phenolic contents *P. mollis* in different solvents with fresh and dried plant parts. *FAE* fresh acetone extract, *FME* fresh methanol extract, *FAqE* fresh aqueous extract, *DAE* dry acetone extract, *DME* dry methanol extract, *DAqE* dry aqueous extract.

Phenolic compounds represent a varied class of plant-derived bioactive secondary metabolites featuring hydroxyl groups (–OH) bonded to aromatic ring systems. These compounds include flavonoids, phenolic acids, coumarins, quinones, stilbenes, and tannins. Folin-Ciocalteu reagent interacts with polyphenols and other reducing agents, resulting in the formation of a blue-coloured complex. This method relies on the ability of phenolic substances to donate electrons to phosphomolybdic and phosphotungstic acid complexes under alkaline conditions. The intensity of the developed blue colouration is quantified at 765 nm using a UV–Visible spectrophotometer. Gallic acid (μg/ml) was used as a standard to construct the calibration curve. The maximum total phenolic content was recorded in dry methanolic leaf extract, measuring 249.9 ± 0.47 mg GAE (Gallic acid equivalent) / g extract, while fresh acetonic root extract shows the lowest phenolic content, 64.12 ± 0.31 mg GAE / g extract (Fig. 5 and Table 2).

**Table 2:** TPC and TFC activity of *P. mollis* Benth.

Extraction code	TPC^α^	TFC^β^
DSAq	158.4 ± 0.63ᵉ	46.60 ± 0.20^b^
DSM	166.5 ± 0.63ᶜ	46.40 ± 0.36^b^
DSA	147.4 ± 0.68ᶠ	43.40 ± 0.19^c^
DLAq	188.7 ± 0.11ᵇ	46.50 ± 0.26^b^
DLM	249.9 ± 0.47ᵃ	42.50 ± 0.20^c^
DLA	162.1 ± 0.34ᵈ	50.50 ± 0.00^a^
DRAq	117.9 ± 0.40ʲ	36.20 ± 0.20^f^
DRM	119.3 ± 0.48ʲ	39.20 ± 0.07^d^
DRA	110.8 ± 0.50ᵏ	37.70 ± 0.00^e^
FSAq	146.0 ± 0.37ᶠ	35.13 ± 0.19^f^
FSM	124.8 ± 0.26ⁱ	30.72 ± 0.34^g^
FSA	102.2 ± 0.23ˡ	28.03 ± 0.23^h^
FLAq	142.4 ± 0.21ᵍ	26.27 ± 0.28^i^
FLM	157.0 ± 0.34ᵉ	28.43 ± 0.23^h^
FLA	132.1 ± 0.31ʰ	36.04 ± 0.27^f^
FRAq	74.68 ± 0.02ⁿ	16.04 ± 0.49^k^
FRM	87.53 ± 0.03ᵐ	16.98 ± 0.45^k^
FRA	64.12 ± 0.31ᵒ	23.17 ± 0.54^j^

*DSAq* dry stem aqueous extract, *DSM* dry stem methanol extract, *DSA* dry stem acetone extract, *DLAq* dry leaf aqueous extract, *DLM* dry leaf methanol extract, *DLA* dry leaf acetone extract, *DRAq* dry root aqueous extract, *DRM* dry root methanol extract, *DRA* dry root acetone extract, *FSAq* fresh stem aqueous extract, *FSM* fresh stem methanol extract, *FSA* fresh stem acetone extract, *FLAq* fresh leaf aqueous extract, *FLM* fresh leaf methanol extract, *FLA* fresh leaf acetone extract, *FRAq* fresh root aqueous extract, *FRM* fresh root methanol extract, *FRA* fresh root acetone extract.

Values are means of three replicate determinations ± standard error. Mean values in the same column with different alphabets shows statistically significant differences (p ≤ 0.05) according to Duncan’s multiple range test. **α** (mg GAE/ g extract) and **β** (mg RE / g extract).

#### Determination of total flavonoid content 

**Fig. 6:**
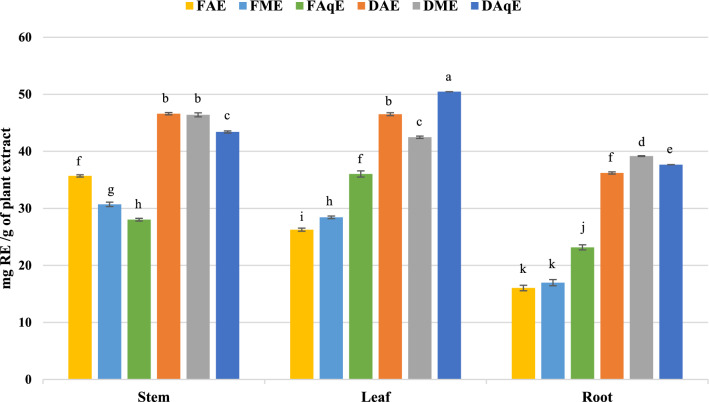
Total flavonoid contents in *P. mollis* in different solvents with fresh and dried plant parts. *FAE* fresh acetone extract, *FME* fresh methanol extract, *FAqE* fresh aqueous extract, *DAE* dry acetone extract, *DME* dry methanol extract, *DAqE* dry aqueous extract.

Flavonoids are composed of two benzene rings (designated as A and B) connected through a three-carbon bridge that forms a central oxygen-containing heterocyclic ring, known as the C ring^26^. Flavonoids can be categorised into various subclasses, including flavonols, flavanones, flavan-3-ols, anthocyanins, flavones, and isoflavones. Flavonoids possess strong antioxidant activity, neutralizing free radicals and minimizing oxidative stress, thereby contributing to the prevention of various chronic illnesses^27^. Flavonoids can exhibit antimicrobial properties by inhibiting the growth of various pathogens^28^. Several flavonoids have shown potential anticancer effects by inducing apoptosis and inhibiting tumor growth^29^. Rutin was employed as the calibration standard for flavonoid quantification. Dry acetonic leaf extract shows the highest total flavonoids content at 50.5 ± 0.00 mg RE (Rutin Equivalents) per gram of extract were the lowest concentration observed in the Fresh aqueous extract of root 16.04 ± 0.49 mg RE/ g of extract (Fig. 6 & Table 2).

### Statistical analysis

**Fig. 7:**
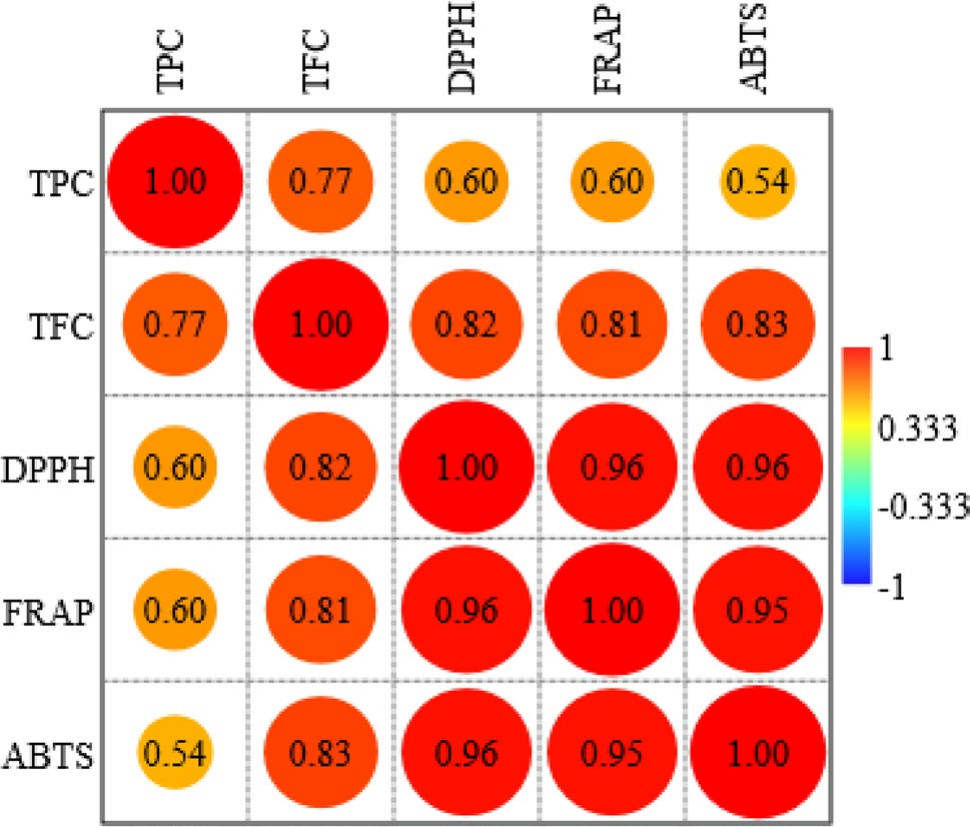
Correlation among various assays, viz. TPC, TFC, DPPH, FRAP, and ABTS (p ≤ 0.05, where p denotes the probability value indicating statistical significance).

The correlation heat map (Fig. 7) presents the Pearson correlation coefficients among total phenolic content (TPC), total flavonoid content (TFC), and three antioxidant assays: DPPH, FRAP, and ABTS. All parameters exhibit positive correlations, with the strongest relationships observed among the antioxidant assays themselves. DPPH shows a very high correlation with both FRAP (r = 0.96) and ABTS (r = 0.96), while FRAP and ABTS are similarly correlated (r = 0.95), indicating consistency and reliability among these methods in evaluating antioxidant activity. TFC is also strongly correlated with the antioxidant assays, showing coefficients of 0.82 (DPPH), 0.81 (FRAP), and 0.83 (ABTS), suggesting that flavonoids significantly contribute to the antioxidant capacity of the plant extracts. In comparison, TPC displays moderate correlations with DPPH (r = 0.60), FRAP (r = 0.60), and ABTS (r = 0.54), indicating a contributory but less dominant role of total phenolics. The moderate correlation between TPC and TFC (r = 0.77) further suggests a partial overlap in their presence within the samples. Overall, the heat map confirms that flavonoid content is more closely associated with antioxidant activity than total phenolic content in the analyzed extracts.

**Fig. 8:**
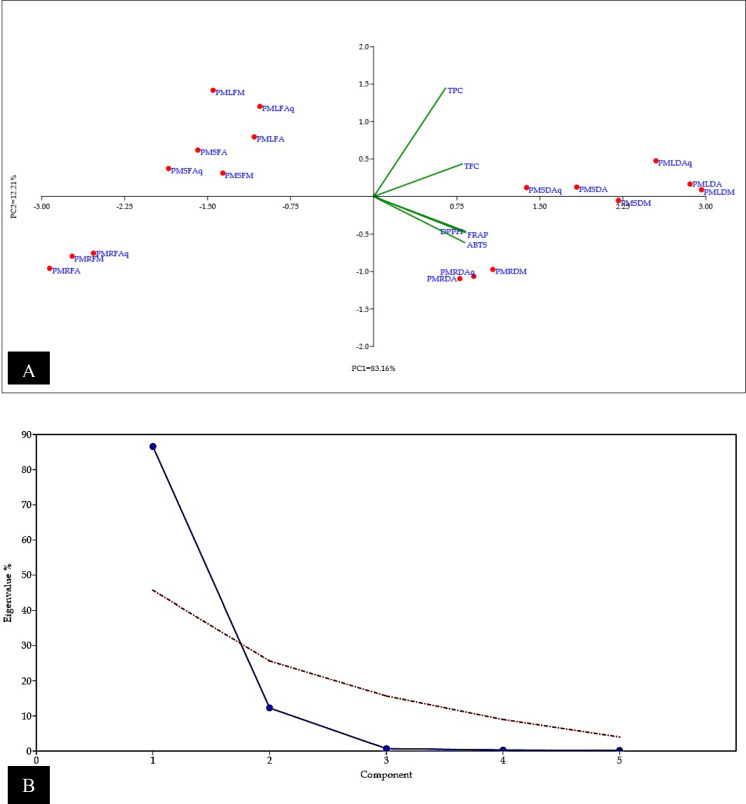
(**A**) Biplot graph based on the principal components analysis (PCA) depicting the phytochemical properties across the *P. mollis*. (**B**) Scree plot showing the percentage of variance explained by the principal components. *DSAq* dry stem aqueous extract, *DSM* dry stem methanol extract, *DSA* dry stem acetone extract, *DLAq* dry leaf aqueous extract, *DLM* dry leaf methanol extract, *DLA* dry leaf acetone extract, *DRAq* dry root aqueous extract, *DRM* dry root methanol extract, *DRA* dry root acetone extract, *FSAq* fresh stem aqueous extract, *FSM* fresh stem methanol extract, *FSA* fresh stem acetone extract, *FLAq* fresh leaf aqueous extract, *FLM* fresh leaf methanol extract, *FLA* fresh leaf acetone extract, *FRAq* fresh root aqueous extract, *FRM* fresh root methanol extract, *FRA* fresh root acetone extract.

The principal component analysis (PCA) biplot (Fig. 8A) shows the distribution of plant extracts based on their phytochemical and antioxidant properties. The first principal component (PC1) accounts for 53.16% of the total variance, while the second principal component (PC2) explains 12.21%, together representing 65.37% of the variability in the dataset. The red-labelled sample points are distributed along the axes, with those positioned on the right side of PC1 (e.g., PMRDA, PMRDM, PMSDA, PMSDAq, PMLDA, and PMLDM) showing a strong positive association with antioxidant assays such as DPPH, FRAP, and ABTS, as indicated by the direction and length of the corresponding vectors. These samples are therefore characterized by high antioxidant activity. In contrast, samples located on the left side of PC1 (e.g., PMRFA, PMRFAq, and PMRFM) are negatively correlated with these variables, suggesting comparatively lower antioxidant potential. The vectors representing total phenolic content (TPC) and total flavonoid content (TFC) are oriented primarily along PC2, indicating their greater influence on the vertical separation of samples. The close alignment of DPPH, FRAP, and ABTS vectors suggests a strong positive correlation among these antioxidant assays. The clustering patterns observed in the biplot reflect distinct biochemical profiles among the extracts, enabling clear discrimination based on their phytochemical content and antioxidant capacity. The scree plot (Fig. 8B) showed a steep drop in the variance explained after the first component. PC1 accounted for nearly 87% of the total variance, while PC2 explained about 12%, and the remaining components (PC3–PC5) contributed insignificantly (≤ 1%). When compared with the broken-stick distribution (46, 28, 20, 14, and 9% for Components 1–5, respectively), only PC1 exceeded its expected threshold, indicating that it is the only component representing meaningful structural variation in the dataset. Therefore, PC1 was considered the principal informative component, whereas the subsequent components were not considered further due to their negligible contribution. The high variance explained by PC1 reflects a robust underlying structure in the dataset, supporting the reliability and interpretability of the PCA results.

**Fig. 9:**
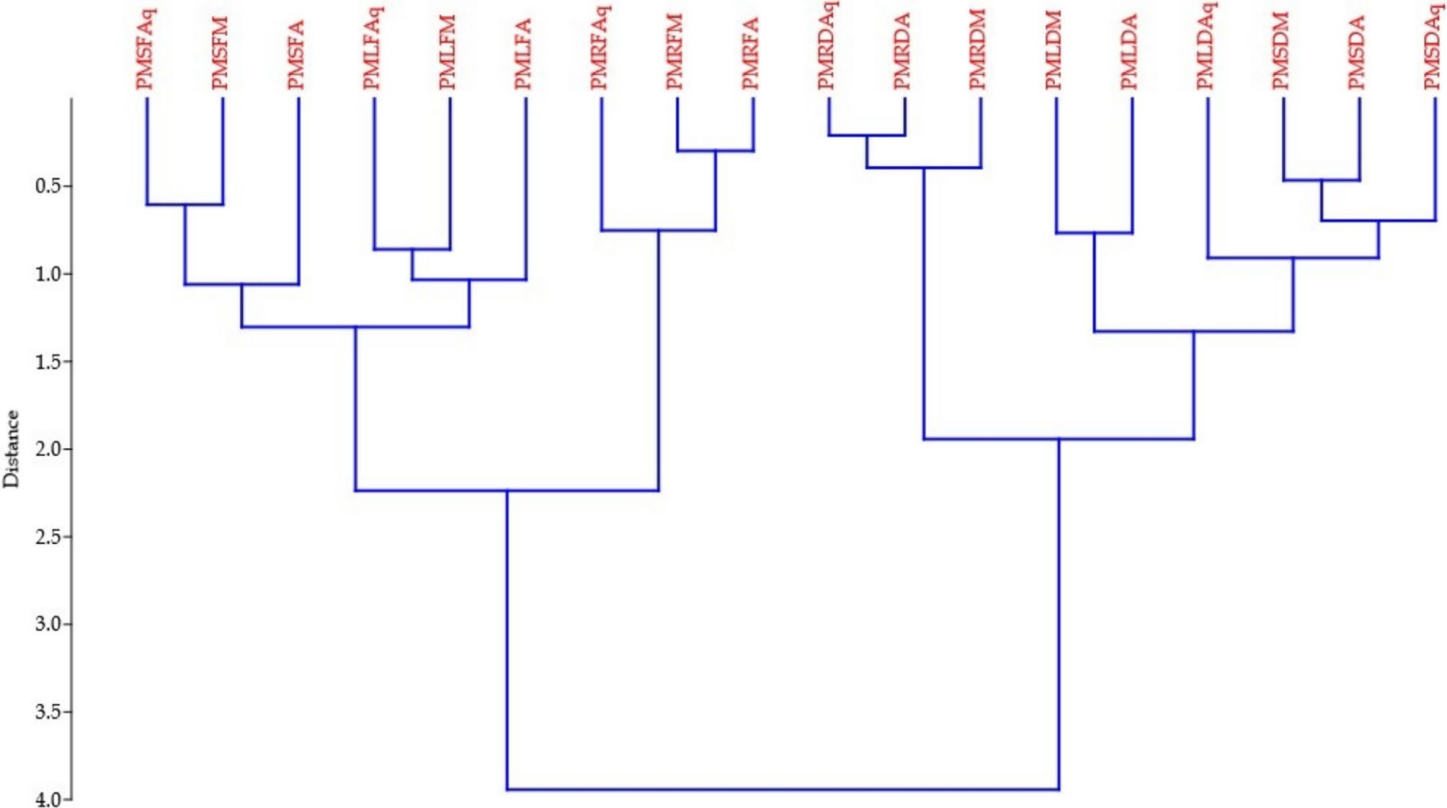
Hierarchical cluster analysis (HCA) of *Pogostemon mollis* extracts based on phytochemical composition and antioxidant parameters.

The hierarchical cluster analysis (HCA) of *Pogostemon mollis* extracts (Fig. 9) demonstrated distinct clustering patterns reflecting variations in chemical composition across solvents and plant parts. Polar extracts, particularly aqueous and methanolic fractions of leaf and stem, formed closely related clusters, indicating high similarity in their phenolic-rich profiles and associated antioxidant activities. In contrast, acetone extracts from all plant parts grouped separately, representing a chemically divergent cluster with lower polarity–derived metabolites. Overall, the HCA clearly differentiates the extracts based on solvent polarity and phytochemical distribution, highlighting the compositional heterogeneity within *P. mollis*.

### Qualitative phytochemical assay (UPLC Q-TOF MS–MS)

**Fig. 10:**
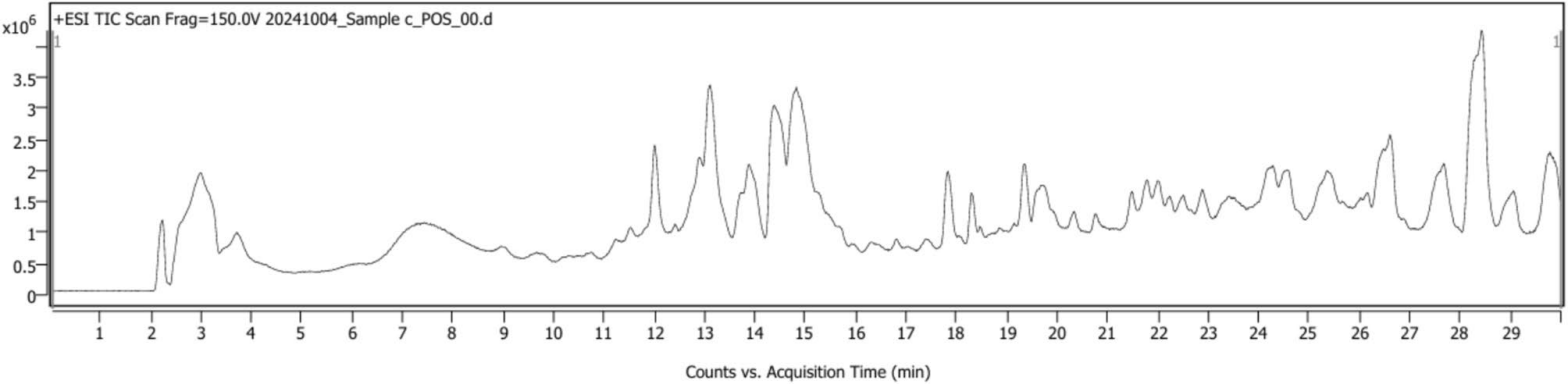
UPLC Q-TOF MS–MS Chromatogram of methanolic extracts of *P. mollis*.

**Fig. 11:**
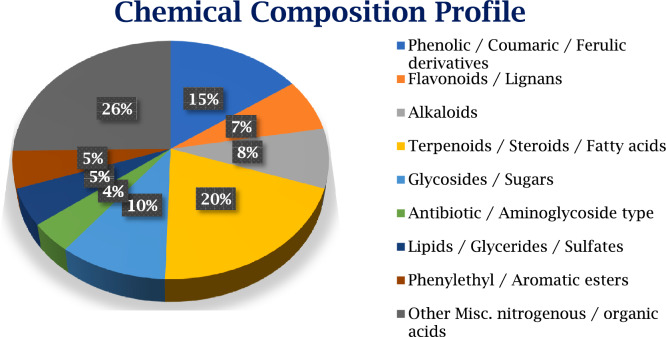
Chemical composition profile of UPLC-Q-TOF–MS/MS-identified metabolites, classified into major phytochemical groups.

**Fig. 12:**
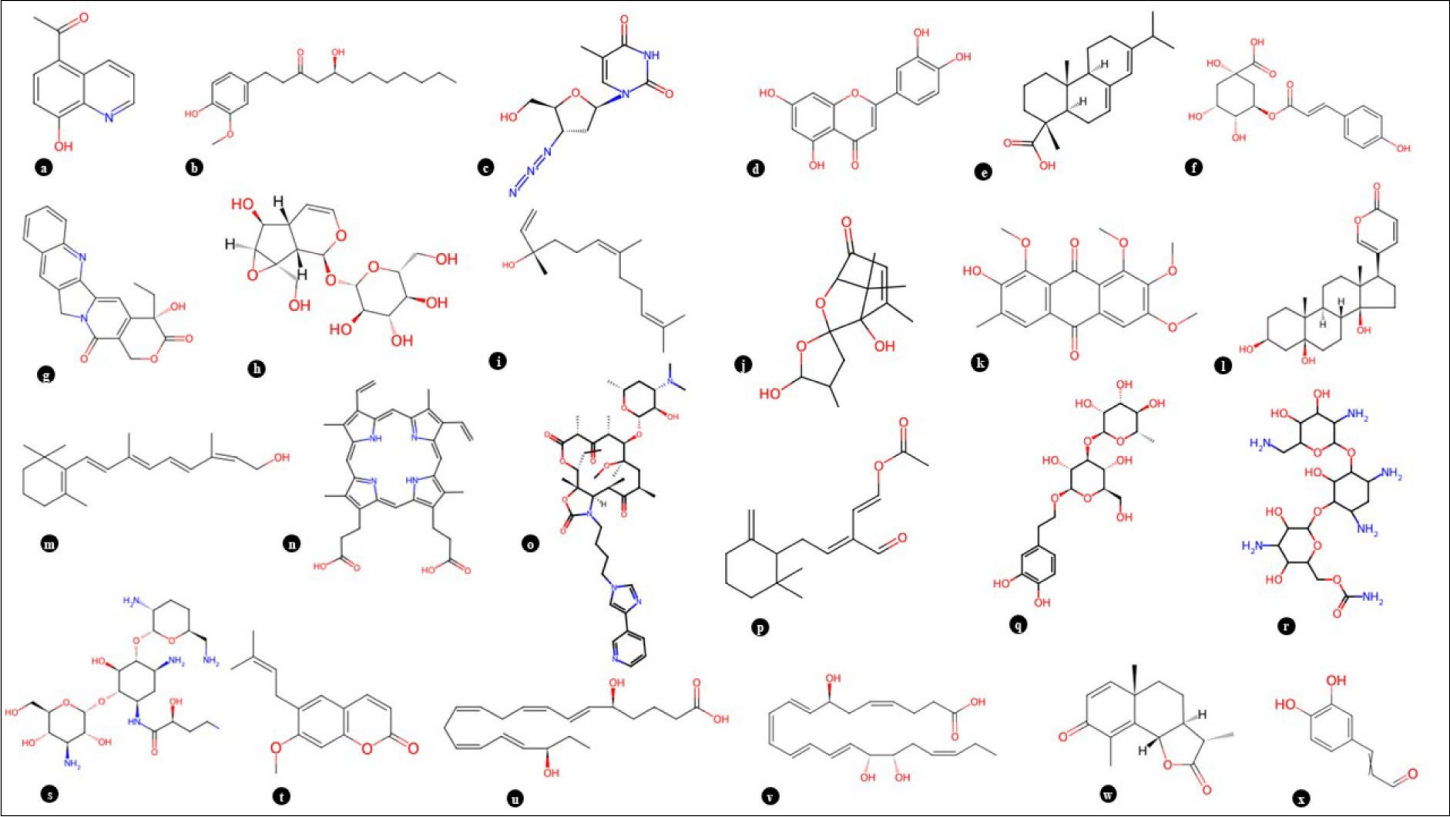
Some important bioactive compounds from the methanolic extract of *P. mollis*. (**a**) Quinacetol, (**b**) 8-Gingerol, (**c**) Zidovudine, (**d**) Luteolin, (**e**) p-Coumaroyl quinic acid, (**f**) p-Coumaroyl quinic acid, (**g**) Camptothecin, (**h**) Catalpol, (**i**) (S)-Nerolidol, (**j**) Kamahine C, (**k**) Chryso-obtusin, (**l**) Telocinobufagin, (**m**) 13-cis-Retinol, (**n**) Protoporphyrin, (**o**) Telithromycin, (**p**) Onchidal, (**q**) Verbasoside, (**r**) Nebramycin factor, 4 (**s**) Arbekacin, (**t**) Suberosin, (**u**) Resolvin E2, (**v**) Resolvin E2, (**w**) α-Santonin, (**x**) Caffeic aldehyde.

UPLC Q-TOF MS–MS is an advanced technique that combines pressurized liquid chromatography and mass spectrometry. It facilitates the separation of components from a compound mixture, while a mass spectrometer produces ions and separates them according to their mass-to-charge ratio. Results indicated the presence of a number of metabolites, including flavonoids, phenolics, toxins, and certain antibiotics. Methanolic extract of *P. mollis* was subjected to liquid chromatography coupled with Mass spectrometry as mentioned in materials and methods. UPLC-QTOF-MS/MS Analysis of *P. mollis* shows 99 compounds (Table 3, Figs. 10, Fig. 11, and Fig. 12). Compounds like the presence of important therapeutic secondary metabolites, including Anticancer Compounds (Camptothecin, Chryso-obtusin, Telithromycin), Antiviral Compounds (Zidovudine, Quinacetol), Phenolics (Sinapic acid), Anti-inflammatory (Resolvin D2, Resolvin E2), Antibiotics (Arbekacin, Nebramycin factor 4), Flavonoids (Luteolin, Chrysosplenetin, Tricetin), Terpenoids (Onchidal, (8)-Gingerol, (S)-Nerolidol), alkaloids (Kamahine C).

**Table 3:** Qualitative analysis of phytochemicals in *P. mollis* using UPLC-Q-TOF–MS analysis.

Sr. no	Compound name	Formula	RT	Mass
1	N-acetyl-D-galactosamine enol	C₁₄H₂₃NO₁₀	2.80	365.1
2	Catalpol	C₁₅H₂₂O₁₀	2.88	362.1
3	1- aminocyclohexanecarboxylic acid	C₇H₁₃NO₂	3.02	143.1
4	Zidovudine	C₁₀H₁₃N₅O₄	3.57	267.1
5	3-hydroxy-cis, cis- muconic acid	C₆H₆O₅	3.73	158.0
6	Trans-4- Carboxymethylenebut-2- en-4-olide	C₆H₄O₄	3.74	140.0
7	Gentiobiosyl 2-methyl-6- oxo-2E,4E-heptadienoat	C₂₀H₃₀O₁₃	8.28	478.2
8	Vanilloloside	C₁₄H₂₀O₈	8.98	316.1
9	Verbasoside	C₂₀H₃₀O₁₂	10.53	462.2
10	1,8- diazacyclotetradecane- 2,9-dione	C₁₂H₂₂N₂O₂	11.04	226.2
11	5,6,7- trimethoxycoumarin	C₁₂H₁₂O₅	11.19	236.1
12	1-O-feruloyl-ß-D-glucos	C₁₆H₂₀O₉	11.22	356.1
13	Quinacetol	C₁₁H₉NO₂	11.52	187.1
14	De-O-methylsimmondsin	C₁₅H₂₃NO₉	11.59	361.1
15	O-1,4-a-L- dihydrostreptosyl- streptidine 6-phosphate	C₁₄H₂₉N₆O₁₁	11.93	488.2
16	6'- methoxypolygoacetophenoside	C₁₅H₂₀O₁₀	12.01	360.1
17	Caffeic aldehyde	C₉H₈O₃	12.02	164.0
18	Sinapic acid	C₁₁H₁₂O₅	12.02	224.1
19	Tuberonic acid glucoside	C₁₈H₂₈O₉	12.03	388.2
20	Luteolin 7-O-[ß-D- glucuronosyl-(1- > 2)-ß D- glucuronide]	C₂₇H₂₆O₁₈	12.57	638.1
21	Phenylethyl primeveroside	C₁₉H₂₈O₁₀	12.80	416.2
22	(7'R) -( +)-lyoniresinol 9’- glucoside	C₂₈H₃₈O₁₃	12.84	582.2
23	Gardoside	C₁₆H₂₂O₁₀	12.92	374.1
24	1-(2,4,5- trimethoxyphenyl)-1,2 propanedione	C₁₂H₁₄O₅	13.07	238.1
25	Fenfuram	C₁₂H₁₁NO₂	13.10	201.1
26	Mahaleboside	C₁₅H₁₆O₈	13.30	324.1
27	Podorhizol beta-D- glucoside	C₂₈H₃₄O₁₃	13.34	578.2
28	5,10- methenyltetrahydrofolate	C₂₀H₂₂N₇O₆	13.53	456.2
29	Arbekacin	C₂₂H₄₄N₆O₁₀	13.58	552.3
30	Luteolin 7-O-glucuronide	C₂₁H₁₈O₁₂	13.71	462.1
31	5-Megastigmen-7-yne- 3,9-diol 9-glucoside	C₁₉H₃₀O₇	14.00	370.2
32	Limonexic acid	C₂₆H₃₀O₁₀	14.09	502.2
33	7-Methyl-1,4,5- naphthalenetriol 4- [xylosyl-(1- > 6)- glucoside]	C₂₂H₂₈O₁₂	15.11	484.2
34	(-)-Matairesinol 4’- [apiosyl-(1- > 2)- glucoside]	C₃₁H₄₀O₁₅	15.11	652.2
35	p-coumaroyl quinic acid	C₁₆H₁₈O₈	15.11	338.1
36	4',5,6- trimethylscutellarein 7- glucoside	C₂₄H₂₆O₁₁	16.23	490.1
37	Chryso-obtusin glucoside	C₂₅H₂₈O₁₂	16.44	520.2
38	Beta-damascenone	C₁₃H₁₈O	16.81	190.1
39	(8)-Gingerol	C₁₉H₃₀O₄	17.07	322.2
40	Camptothecin	C₂₀H₁₆N₂O₄	17.19	348.1
41	9S,11R,15S-trihydroxy- 2,3-dinor-13E- prostaenoic acid- cyclo[8S,12R]	C₁₈H₃₂O₅	17.41	328.2
42	Myristic acid	C₁₄H₂₈O₂	17.46	228.2
43	5-Hydroxy-1-(4-hydroxyphenyl)-3- decanone	C₁₆H₂₄O₃	17.70	264.2
44	Trinexapac-ethyl	C₁₃H₁₆O₅	17.88	252.1
45	9,10-Dihydroxy-12,13- epoxyoctadecanoate	C₁₈H₃₄O₅	18.09	330.2
46	Abietic acid	C₂₀H₃₀O₂	18.49	302.2
47	Resolvin D2	C₂₂H₃₂O₅	19.15	376.2
48	Heptopargil	C₁₃H₁₉NO	19.23	205.1
49	3',4',5'-Trimethoxycinnamyl alcohol acetate	C₁₄H₁₈O₅	19.36	266.1
50	Phytosphingosine	C₁₈H₃₉NO₃	19.81	317.3
51	16-Hydroxy hexadecanoic acid	C₁₆H₃₂O₃	19.91	272.2
52	Suberosin	C₁₅H₁₆O₃	19.95	244.1
53	α-santonin	C₁₅H₁₈O₃	20.33	246.1
54	(2E,4E,6E)-2,6 dimethylocta-2,4,6 trienedial	C₁₀H₁₂O₂	20.34	164.1
55	Resolvin E2	C₂₀H₃₀O₄	20.35	334.2
56	Chrysosplenetin	C₁₉H₁₈O₈	20.46	374.1
57	5-O-Methylvisamminol	C₁₆H₁₈O₅	20.91	290.1
58	11beta,17beta Dihydroxy-17-methyl 5alpha-androstan-3-one	C₂₀H₃₂O₃	21.17	320.2
59	Normethandrolone	C₁₉H₂₈O₂	21.54	288.2
60	(S)-nerolidol 3-O-[a-L rhamnopyranosyl-(1 > 4)-a-L rhamnopyranosyl-(1 > 2)-b-D glucopyranoside]	C₃₃H₅₆O₁₄	21.55	676.4
61	Kamahine C	C₁₄H₂₀O₅	21.59	268.1
62	Tricetin 3’,4',5'-trimethyl ether	C₁₈H₁₆O₇	21.71	344.1
63	Sphinganine	C₁₈H₃₉NO₂	21.72	301.3
64	13-cis-retinol	C₂₀H₃₀O	21.79	286.2
65	10-hydroperoxy-8E,12Z octadecadienoic acid	C₁₈H₃₂O₄	21.80	312.2
66	Colneleic acid	C₁₈H₃₀O₃	21.80	294.2
67	(S)-nerolidol 3-O-[a-L rhamnopyranosyl-(1 > 4)-a-L rhamnopyranosyl-(1 > 2)-b-D glucopyranoside]	C₃₃H₅₆O₁₄	21.96	676.4
68	Hostmaniane	C₁₃H₁₈O₅	22.53	254.1
69	3alpha,21-dihydroxy-D homo-5beta-pregn 17a(20)-en-11-one	C₂₂H₃₄O₃	22.65	346.3
70	Onchidal	C₁₇H₂₄O₃	22.89	276.2
71	Gingerglycolipid B	C₃₃H₅₈O₁₄	23.28	678.4
72	3-α(S)-strictosidine	C₂₇H₃₄N₂O₉	23.44	530.2
73	Chryso-obtusin	C₁₉H₁₈O₇	23.56	358.1
74	Protomycinolide IV	C₂₁H₃₂O₄	23.68	348.2
75	16-feruloyloxypalmitate	C₂₆H₄₀O₆	23.73	448.3
76	Lauryl hydrogen sulfate	C₁₂H₂₆O₄S	23.91	266.2
77	Isopimara-7,15-dienol	C₂₀H₃₂O	24.03	288.2
78	Aspidinol	C₁₂H₁₆O₄	24.09	224.1
79	2alpha hydroxypyracrenic acid	C₃₉H₅₄O₇	24.11	634.4
80	3-α(S)-strictosidine	C₂₇H₃₄N₂O₉	24.14	530.2
81	3-O-trans feruloyleuscaphic acid	C₄₀H₅₆O₈	24.35	664.4
82	Telocinobufagin	C₂₄H₃₄O₅	24.43	402.2
83	Nebramycin factor 4	C₁₉H₃₈N₆O₁₁	24.91	526.3
84	Beta-elemonic acid	C₃₀H₄₆O₃	25.14	454.3
85	Sterol 3-beta-D glucoside	C₂₃H₃₈O₆	25.35	410.3
86	F4-Neuroprostane (7 series)	C₂₂H₃₄O₅	25.41	378.2
87	Colneleic acid	C₁₈H₃₀O₃	25.42	294.2
88	Isopimara-7,15-dienol	C₂₀H₃₂O	25.47	288.2
89	Di-n-heptyl phthalate	C₂₂H₃₄O₄	25.64	362.2
90	3-oxo-5alpha-steroid	C₂₂H₃₆O₃	26.54	348.3
91	Telithromycin	C₄₃H₆₅N₅O₁₀	27.71	811.5
92	1-palmitoyl lysophosphatidic acid	C₁₉H₃₉O₇P	27.71	410.2
93	Protoporphyrin	C₃₄H₃₄N₄O₄	27.72	562.3
94	Dihydrozeatin-9-N glucoside-O-glucoside	C₂₂H₃₅N₅O₁₁	27.78	545.2
95	2-undecyl-4(1H) quinolinone N-oxide	C₂₀H₂₈NO₂	28.36	314.2
96	Di-n-heptyl phthalate	C_22_ H_34_ O_4_	28.67	362.2
97	Polidocanol	C₃₀H₆₂O₁₀	28.79	582.4
98	Dihydroabietic acid	C₂₀H₃₂O₂	28.98	304.2
99	Integerressine	C₃₃H₃₈N₄O₄	29.24	554.3

### Essential oil profiling (GC–MS)

Gas chromatography relies on chromatograms, which are printouts displaying peaks representing different components of an essential oil, to separate substances. However, it cannot directly identify the substance responsible for a peak. Instead, comparison with known standards is necessary to make identifications. If a known standard appears at the same position as a peak in the chromatogram, the substance is assumed to be identified. Identifying components in essential oils can be challenging due to their complex compositions. To achieve precise identification, spectroscopic methods are often necessary. Recent advancements have simplified this process with the introduction of machines capable of conducting mass spectrometry immediately after gas chromatography separation. These machines perform a dual function within a single unit: first, separating the essential oil components via gas chromatography, and then individually identifying the isolated components through separate mass spectra. Each compound produces its own spectrum. However, interpreting these mass spectra is a complex process and is primarily facilitated by computerized reference libraries.

**Fig. 13:**
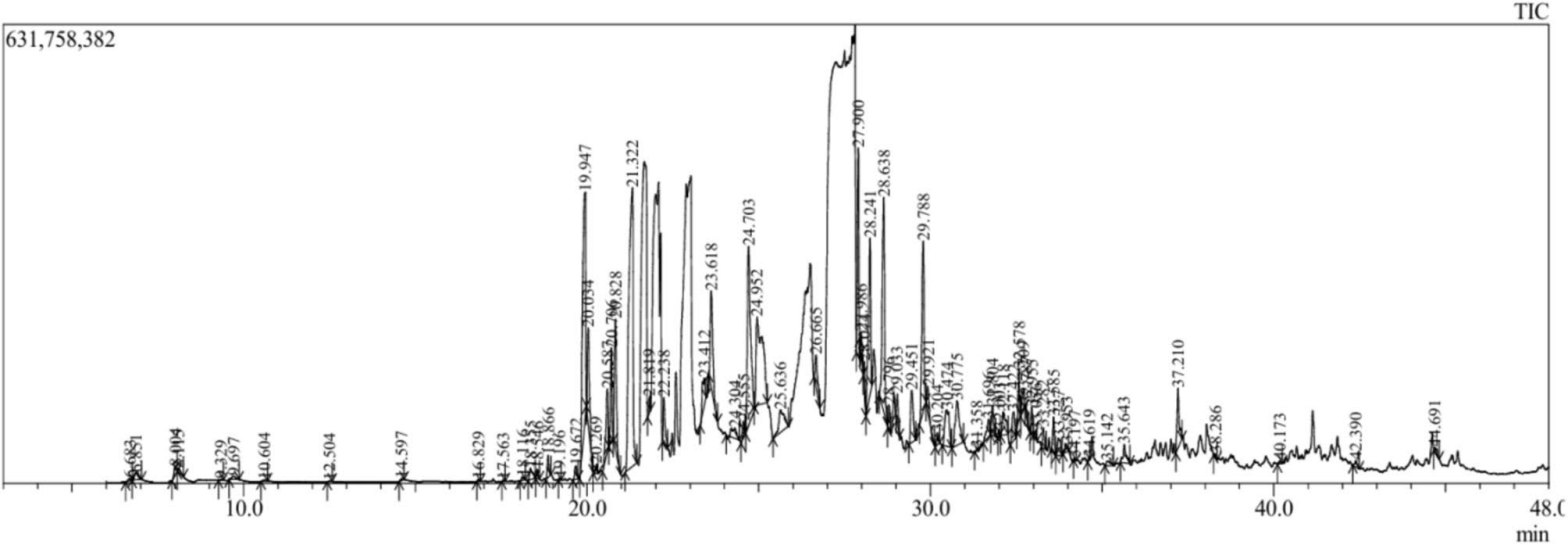
GC–MS Chromatogram of Essential oil of *P. mollis*.

**Fig. 14:**
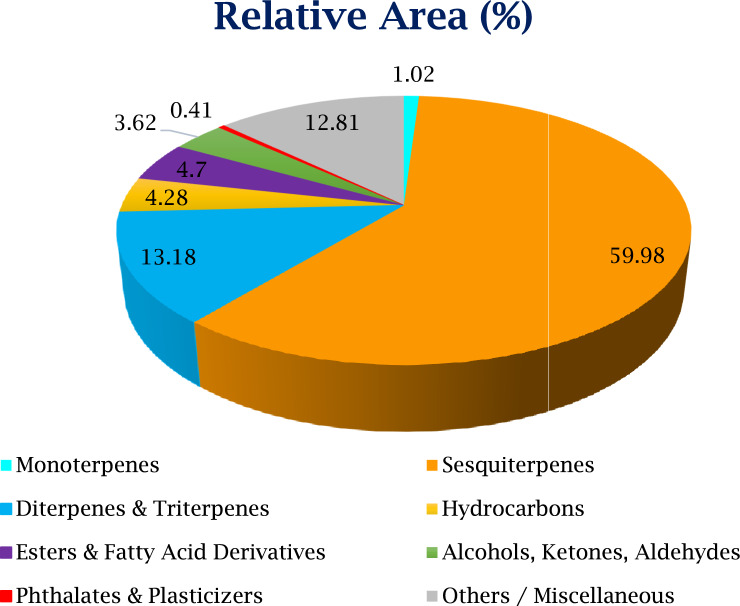
GC–MS-based profiling of major compound classes in *Pogostemon mollis* essential oil.

**Fig. 15:**
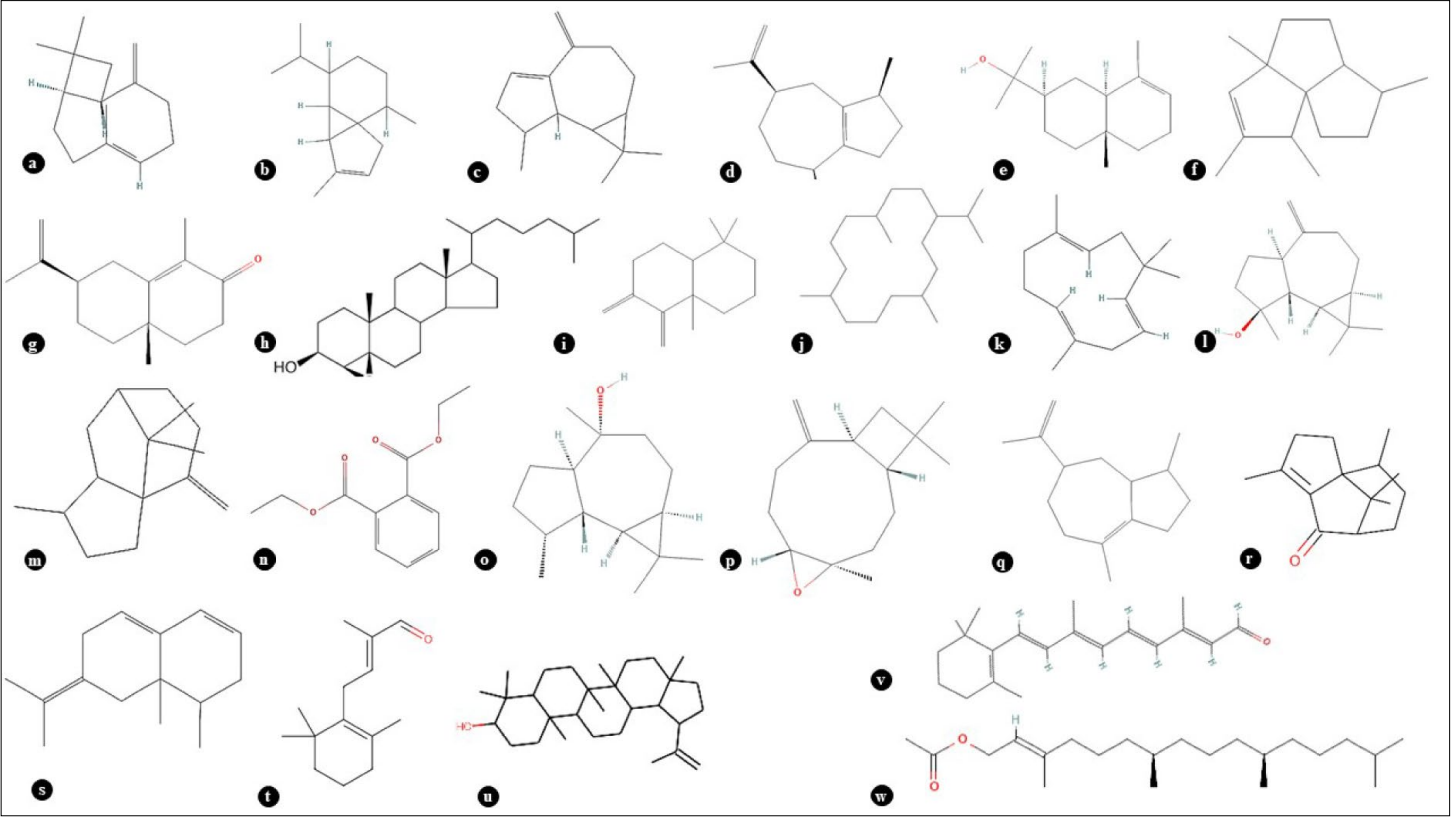
Some important bioactive compounds from the essential oil of *P. mollis*. (**a**) Caryophyllene, (**b**) (-)-alpha-Cubebene, (**c**) Aromadendrene, (**d**) alpha-Guaiene, (**e**) Alpha-eudesmol, (**f**) 7-epi-Silphiperfol-5-ene, (**g**) Alpha-Cyperone, (**h**) 3α,7β-Dihydroxy-5β,6β-epoxycholestane, (**i**) 1,1,4a-Trimethyl-5,6-dimethylenedecahydronaphthalene, (**j**) Cembrane, (**k**) Humulene, (**l**) Spathulenol, (**m**) Gamma-Patchoulene, (**n**) Diethyl Phthalate, (**o**) Globulol, (**p**) Caryophyllene oxide, (**q**) Guaia-1(10),11-diene, (**r**) Isopatchoulenone, (**s**) beta-Vatirenene, (**t**) Boronal, (**u**) Lupeol, (**v**) Retinal, (**w**) Phytyl acetate.

The oil from *P. mollis* was pale yellow in colour. GC–MS of the essential oil of *P. mollis* showing the presence of a total of 68 compounds. The essential oil yield (0.6%) is in this species. This compound list was given in (Table 4, Figs. 13, Fig. 14 & Fig. 15). The maximum representative compounds from *P. mollis* were lupeol (6.33%), alpha. -cyperone (7.15%), t (7.23%), globulol (5.07%), boronal (7.51%), guaia-10 (14),11-diene (2.31%) and 7-epi-Silphiperfol-5-ene (9.46%). Previous studies have highlighted compounds like cadina-1,4-diene (26.64%), sabinene (11.95%), beta-pinene (8.34%), beta-cubebene (7.76%), and alpha-pinene (5.95%)^30^. Caryophyllene oxide (CO), a bioactive sesquiterpene, is used in cancer treatment^31^.

**Table 4:** Essential oil profiling of *P. mollis* by GC–MS/MS.

Peak no	Compound name	RT	Area%
1.	Alpha-thujene	6.683	0.08
2.	Alpha-Pinene	6.851	0.31
3.	Sabinene	8.004	0.22
4.	Beta-pinene	8.115	0.11
5.	Cis-4-carene	9.329	0.04
6.	D-Limonene	9.697	0.16
7.	Gamma-terpinene	10.604	0.04
8.	3-methylcyclopentenone	12.504	0.02
9.	4-terpinenol	14.597	0.06
10.	2,6,11-trimethyldodecane	16.829	0.04
11.	Behenyl behenate	17.563	0.02
12.	Isopentacosane	18.116	0.09
13.	Alpha. -eudesmol	18.355	0.43
14.	Delta. -Eiemene	18.546	0.19
15.	Alpha.—cubebene	18.866	0.46
16.	Gamma.—muurolene	19.196	0.03
17.	4,4-dimethyl-3-(3-methylbut-3-enylidene)-2-methylenebicyclo [4.1.0] heptane	19.672	0.36
18.	7-epi-silphiperfol-5-ene	19.947	9.46
19.	Guaia-10(14),11-diene	20.034	2.31
20.	Tetradecane	20.269	0.16
21.	Caryophyllene	20.587	1.65
22.	1,1,4a-trimethyl-5,6-dimethylene-decalin	20.706	1.82
23.	Aromandendrene	20.828	3.45
24.	Gamma-patchoulene	21.322	14.91
25.	Humulene	21.819	0.20
26.	Alpha.—guaiene	22.238	1.10
27.	Beta.—vatirenene	23.412	1.13
28.	Isovalencenol	23.618	2.97
29.	Globulol	24.304	5.07
30.	4,10-aromadendranediol	24.555	0.30
31.	Boronal	24.703	7.51
32.	Caryophyllene oxide	24.952	7.23
33.	Diethyl phthalate	25.636	1.58
34.	Spathulenol	26.665	1.04
35.	Acetic acid, 3-hydroxy-6-isopropenyl-4,8a-dimethyl-1,2,3,5,6,7,8,8a-octahydronaphthalen-2-yl ester	27.900	4.12
36.	Eudesma-4,11-dien-2-ol	27.986	0.22
37.	Cembrane	28.074	0.36
38.	Lupeol	28.638	6.33
39.	Isopatchoulenone	28.796	0.41
40.	Allopregnanolone	29.033	0.59
41.	Retinal	29.451	1.82
42.	Alpha. -cyperone	29.788	7.15
43.	E, E, Z-1,3,12-nonadecatriene-5,14-diol	29.921	0.46
44.	Aristolone	30.204	0.27
45.	3alpha,7beta-dihydroxy-5beta,6beta-epoxycholestane	30.775	2.37
46.	Phytane	31.358	0.01
47.	2-butyloxycarbonyloxy-1,1,10-trimethyl-6,9-epidioxydecalin	31.696	0.65
48.	1,54-dibromotetrapentacontane	31.804	1.22
49.	Eicosane	32.003	0.06
50.	Phytyl acetate	32.118	1.06
51.	Diglycolic acid	32.422	0.95
52.	Carbonic acid	32.578	0.97
53.	Corymbolone	32.709	0.59
54.	Diene-2,8-dione	32.787	0.14
55.	Methyl palmitate	33.056	0.10
56.	Hexacosyl nonyl ether	33.282	0.53
57.	Hexadecane	33.585	0.73
58.	Tetrapentacontane	33.727	0.71
59.	Butyl isodecyl phthalate	33.953	0.27
60.	Ethyl palmitate	34.619	0.04
61.	2,3-dimethylnonadecane	35.142	0.24
62.	Dotriacontane	35.643	0.73
63.	Hexacontane	37.210	1.61
64.	2-methylhexacosane	38.286	0.05
65.	Pentatriacontane	40.173	0.08
66.	Tetracosane	42.390	0.19
67.	Phthalic acid	44.691	0.14
68.	Squalene	49.910	0.28

## Conclusion

This study provides a comprehensive investigation into phytochemical composition, essential oil profile, and antioxidant potential of *P. mollis* collected from Trivandrum district, Kerala. Antioxidant activity assessed through DPPH, FRAP, and ABTS assays revealed notable free radical scavenging and reducing capacities, particularly evident in dry aqueous and methanolic extracts. Biochemical analysis revealed substantial variability in total phenolic and flavonoid contents across different extracts, with dry methanolic leaf extracts exhibiting the highest TPC and TFC values. UPLC-QTOF-MS/MS analysis identified 99 bioactive metabolites, including pharmacologically relevant compounds like camptothecin, zidovudine, and luteolin, while GC–MS analysis identified 68 volatile constituents, with notable compounds such as lupeol, alpha-cyperone, and caryophyllene. Study revealed a strong statistical link between phenolic and flavonoid contents and antioxidant activity of *P. mollis* may serve as an effective natural antioxidant source. These findings emphasize the potential utility of this species in formulating bioactive compounds and validate its traditional use in herbal medicine for health-promoting purposes. Future studies, including in vivo investigations and bioactivity-guided fractionation, are warranted to further elucidate the pharmacological potential, therapeutic applicability of *P. mollis*.

## Data Availability

The datasets generated and analysed during the current study are available from the corresponding author on reasonable request.
